# Effects of Elevated CO_2_ and Increased N Fertilization on Plant Secondary Metabolites and Chewing Insect Fitness

**DOI:** 10.3389/fpls.2019.00739

**Published:** 2019-06-04

**Authors:** Huaping Xu, Haicui Xie, Shengyong Wu, Zhenying Wang, Kanglai He

**Affiliations:** ^1^College of Agronomy and Biotechnology, Hebei Normal University of Science and Technology, Qinhuangdao, China; ^2^The State Key Laboratory for Biology of Plant Diseases and Insect Pests, Institute of Plant Protection, Chinese Academy of Agricultural Sciences, Beijing, China

**Keywords:** climate change, elevated CO_2_, N fertilization, plant-insect interaction, metabolites

## Abstract

Elevated atmospheric CO_2_ (eCO_2_) and increased nitrogen (N) fertilization significantly change the nutritional quality of plants and influence the growth and development of insects. However, little is known about plant metabolism and plant-insect interactions under eCO_2_ and increased N fertilization, especially C_4_ plants. Thus, the combined effects of eCO_2_ and increased N fertilization on maize-*Ostrinia furnacalis* interactions were tested in this study. Our data demonstrated that both eCO_2_ and increased N fertilization increased starch content, while increased N fertilization promoted the N content in maize. The combined effects of eCO_2_ and increased N fertilization did not influence the total non-structural carbohydrates (TNC):N ratio in maize. The jasmonic acid level of maize was enhanced by increased N fertilization and *O. furnacalis* infestation. The total phenolics content and defensive enzyme activities of maize increased under eCO_2_, increased N fertilization and *O. furnacalis* infestation. Protective enzyme activities were enhanced, while digestive enzyme activities, mean relative growth rate, body mass and efficiency of conversion of ingested food decreased for *O. furnacalis* feeding on maize grown under eCO_2_ and increased N fertilization. Therefore, eCO_2_ and increased N fertilization increased starch and N accumulation, and did not influence the TNC:N ratio, however, eCO_2_ and N promoted the resistance-related secondary metabolites (with or without *O. furnacalis* induced) of maize, which ultimately decreased the fitness of *O. furnacalis* to the host. These results will help to better understand the metabolic mechanisms of plants and the plant-insect interaction under eCO_2_ and increased N fertilization in the context of future climate change scenarios.

## Introduction

Industrialization has increased atmospheric CO_2_ concentration from 280 ppm at the beginning of the Industrial Revolution to 400 ppm today and the value is expected to double by the end of the century (IPCC, 2014). Generally, carbohydrate content in plants increases with elevated atmospheric CO_2_ (eCO_2_), due to higher photosynthetic rates (Long et al., 2006; Reddy et al., 2010; Kimball, 2016). Plants grown in eCO_2_ are observed to have lower nitrogen (N) concentration compared to plants grown in ambient CO_2_. However, the interpretations for lower N concentration are inconsistent across the literatures. There exist several reasons for this: the dilution effect due to enhanced production of carbohydrates (Novriyanti et al., 2012), the decrease in the specific uptake rates of N by roots under eCO_2_ (Taub and Wang, 2008), or that eCO_2_ directly inhibits plant nitrogen metabolism (Bloom et al., 2012). Therefore, more sophisticated approaches to nitrogen fertilization should be employed to enhance N concentration and improve plant quality under eCO_2_ (Bloom et al., 2014).

Present and future eCO_2_ is predicted to have a profound effect on plant-insect interactions due to changes in the C:N ratio and chemical compositions based on primary and secondary metabolites (Zavala et al., 2017). This change impacts the food quality of the host plant which subsequently influences insect growth and development. For example, eCO_2_ increases the total non-structural carbohydrates (TNC) content in maize plants and decreases the N content, causing insects to consume more plant tissue to obtain enough N-based nutrients, and extends their development time (Yin et al., 2010; Xie et al., 2015). At the same time, eCO_2_ also influences plant secondary metabolites that affect insect growth and development (Reddy et al., 2004; Chen et al., 2005b). For example, under eCO_2_, the contents of tannin and gossypol are increased and Bt protein synthesis is decreased in cotton (Chen et al., 2005b), the content of total phenolics is increased in rape seed (Reddy et al., 2004), and the defensive enzyme activities against aphids are decreased in *Medicago truncatula* (Guo et al., 2014).

Increased N fertilization is expected to enhance the N content in plants grown under eCO_2_. Previous studies have shown that increased N fertilization of wheat plants increases nitrate reductase activity, whereas eCO_2_ reduces it, and the combined effects result in additional protein content (Pal et al., 2005; Xu et al., 2011). However, Sudderth et al. (2005) indicated that higher N availability in the presence of eCO_2_ increased foliar N content in *Amaranthus viridis* (C_4_ plant), but had no effect on *Solanum dulcamara* (C_3_ plant). Therefore, we reason that the responses of C_3_ and C_4_ plants to increased N fertilization under eCO_2_ are entirely different.

Several studies have indicated that the availability of N fertilization determines plant allocation of defensive compounds in response to eCO_2_, which subsequently alters insect performance (Kinney et al., 1997; Hättenschwiler and Schafellner, 1999; Henn and Schopf, 2001; Saxon et al., 2004). However, changes in nutritional and defensive metabolisms of plants grown under eCO_2_ and increased N fertilization, as well as insect fitness remain unclear.

Plants have evolved a series of defense systems against insects, one of which is the jasmonic acid (JA) signaling pathway (Kawazu et al., 2012). Several studies have examined the insect-induced plant defense response via JA under eCO_2_, however, these responses have varied for different plant and insect species (Casteel et al., 2008; Zavala et al., 2008; Lu et al., 2018). In view of the above-mentioned facts, even if N fertilization increases the protein content in plant tissue under eCO_2_, it is not clear if this will be able to influence JA-mediated secondary metabolites.

Asian corn borer, *Ostrinia furnacalis* (order Lepidopteran), consumes C_4_ plant-maize. One to seven generations of this insect occur per year in northern, cool temperate regions to southern tropical areas (Zhou et al., 1995). They cause a 10–30% yield loss in most maize production areas in China (Wang et al., 2000). Our previous study showed that the fitness of *O. furnacalis* in maize was adversely affected by an eCO_2_-mediated decrease in maize nutritional quality (higher TNC:N ratio) during a field experiment (Xie et al., 2015). Based on this result and the evidence for different responses of C_3_ and C_4_ plants to increased N fertilization under eCO_2_, as mentioned above, the interactions between maize and *O. furnacalis* may change due to the combined effects of eCO_2_ and increased N fertilization.

The specific objectives of this study were to (1) quantify the effects of eCO_2_ and increased N fertilization in maize on C and N primary metabolites and *O. furnacalis* induced defense-related secondary metabolites, (2) determine the successive performance of *O. furnacalis* to the host. Results from our study help to understand the metabolic mechanism of the plant and the plant-insect interactions under eCO_2_ and increased N fertilization, which will subsequently aid in adjusting pest control strategies under the context of future climate change.

## Materials and Methods

### Plant Preparation and Treatments With CO_2_ and Nitrogen

The experiment was carried out in six environmental chambers with three environmental chambers maintained at ambient CO_2_ concentrations (∼380 ppm) and three environmental chambers maintained at elevated CO_2_ concentrations (∼750 ± 15 ppm). The ambient CO_2_ was the surrounding air entering the environmental chamber facilities, whereas the elevated CO_2_ was supplied from direct gas tanks. Details of the automatic control system for CO_2_ levels were described by Chen et al. (2005a). The environmental chambers were maintained at 28 ± 1°C and 60–70% RH, with a photoperiod of 16:8 h (L:D) and illumination (90 μmol m^−2^ s^−1^) provided by fluorescent lamp. Maize seeds were planted in trays (26.5 cm × 18.5 cm × 7.5 cm) filled with a sand, peat and vermiculite mixture (1:1:1). Three N fertilization levels were established, low N (100 mg N/kg soil mixture); middle N (200 mg N/kg soil mixture), and high N (300 mg N/kg soil mixture) under each CO_2_ level (i.e., in each environmental chamber). In preliminary experiments, low N produced adequate growth of maize plants, while high N significantly enhanced growth. Each tray was planted with 20 seedlings, three trays were used in each N fertilization level. Of these three trays, maize plants from two trays were used for *O. furnacalis* rearing experiment, and from the remaining tray was used for metabolic analysis. After collecting tissue for metabolic analysis, the tray with maize plants was removed from the environmental chamber to avoid the wounding signals transmission among the plants. All trays were provided the same amount of water (100 mL) each day. To account for possible spatial variability from micro environmental factors, the environmental chambers were arranged in three blocks of two adjacent chambers per block.

### Insect Rearing

The *O. furnacalis* neonates used in this study were obtained from a laboratory colony that originated from a field population and had been maintained on a regular artificial diet (Zhou et al., 1995) for 5–6 generations in the lab. The centrifuge tubes (50 mL) were used as *O. furnacalis* larval rearing containers in the environment chamber as above description. Centrifugal tube orifice was covered with three layers of gauze. A piece of maize leaf (1 cm × 4 cm) from the same environment chamber was placed in a centrifuge tube and fed by a neonate larva (∼12 h). When larva developed to the 4th instar, a short stem (5 cm long) was supplied to it instead of leaf tissue (Zhou et al., 1995). Twenty larvae were reared in each treatment, with three replications per treatment (total 60 larvae per treatment). The leaf or stem issues were replaced with fresh leaves or stems every other day until larvae pupated. Meanwhile the unconsumed leaf- or stem-tissues and frass were collected and weighed after oven drying at 80°C for 72 h. The water content was calculated by daily drying of the fresh leaf or stem to obtain the dry weight of larval food consumption. The neonate (∼12 h) and mature larvae (before pupation) fresh weight were measured and the duration (in days) of larval and pupal stages were also recorded. The conventional, ratio-based nutritional indices, including mean relative growth rate (MRGR), efficiency of conversion of ingested food (ECI) and of digested diet (ECD), and approximate digestibility (AD) were determined gravimetrically following the methods of Rayapuram and Baldwin (2006) and Chen et al. (2007). The amount of food consumption, frass produced, larval body weight, and weight gain were all calculated as dry weights. Formulas for calculation of the indices measured are shown in Table 1.

**Table 1:** Calculations for indices for larval growth, development, and food digestibility.

Measuring indices	Formulation
Mean relative growth rate, MRGR	MRGR = (logW_2_−logW_1_)/t
Approximate digestibility (%), AD	AD = [(Q−F)/Q]^∗^100
Efficiency of conversion of ingested food (%), ECI	ECI = [(W_2_−W_1_)/Q]^∗^100
Efficiency of conversion of digested food (%), ECD	ECD = [(W_2_−W_1_)/(Q-F)]^∗^100

*^∗^Q, the diet consumption (mg); F, the frass excretion (mg); W_1_, the initial larval wet mass (mg), W_2_, the final larval wet body mass (mg); t, days from the initial to the final day (d).*

The *O. furnacalis* feeding maize plant from each treatment were used to test the activity of digestive and protective enzymes. The digestive enzymes included trypsin, total protease, lipase and amylase, the protective enzymes included catalase (CAT), peroxidase (POD), and superoxide dismutase (SOD). 10 larvae (4–5th instar) from each treatment were selected and were grinded in physiological saline (0.9%). Homogenates were centrifuged at 10,000 × *g* for 10 min, and the supernatants were subjected to enzyme activity analysis according to the kit instructions (MM-126401 for Trypsin, MM-3439701 for total protease, MM-3442501 for lipase, MM-3439801 for amylase, MM-3440701 for CAT, MM-3440801 for POD, MM-3439601 for SOD, Jiangsu Kete Biological Technology Co., Ltd., China).

### Primary and Secondary Metabolites Analysis of Maize

For primary metabolic analysis, one leaf was collected at random from each maize plant on the 20th day after sowing, a total of 5 leaves were collected in each tray and mixed for leaf samples. The TNC (primarily soluble sugars and starch) were analyzed using the method of Tissue and Wright (1995). The nitrogen content was determined according to the Official Methods of Analysis of AOAC International (2009), using a Kjeltec N analyzer (Model KDY-9830; Foss automated Kjeltec instruments, Beijing, China).

For analysis of secondary defense metabolism, 2nd to 3rd instar larvae of *O. furnacalis* were placed on each maize leave. Damaged areas from approximately 1 cm around the feeding sites were taken from the leaves using a knife. Leaf samples were collected 2 h after infestation (Guo et al., 2018). One leaf was collected at random from each maize plant on the 20th day after sowing, a total of 5 leaves were harvested in each tray and mixed for leaf samples. After harvesting, leaf samples were immediately frozen in liquid nitrogen and then stored at −80°C until use. Uninfested leaves were used as controls and treated as described above. For JA analysis, frozen leaves (0.1 g) were ground in 1:10 [leaf: extracting solution (w/v) ratio]. The extracting solution contained methyl alcohol, formic acid, and pure water (15/1/4, v/v/v). Homogenates were centrifuged at 8,000 × *g* for 20 min. The supernatants were subjected to JA content analysis according to the kit instructions (MM-0887001, Keteshengwu, Nanjing, China). For the remaining analysis, frozen leaves (0.1 g) were ground in buffer (1:10 w/v ratio; pH 7.0–7.4). Homogenates were centrifuged at 10,000 × *g* for 10 min. The supernatants were then analyzed for total phenolics content, the activities of polyphenol oxidase (PPO), POD, phenylalanine ammonia-lyase (PAL), CAT, and proteinase inhibitors (PIs) according to the kit instructions (MM-123301 for total phenolics, MM-3269901 for PPO, MM-3596001 for POD, MM-089901 for PAL, MM-079901 for CAT, MM-063301 for PIs, Jiangsu Kete Biological Technology Co., Ltd., China).

### Statistical Analyses

The main effects of CO_2_, N, infestation and block (pairs of adjacent environmental chambers) on plant secondary defense metabolism (JA and total phenolics content; and PPO, POD, CAT, PAL, and PIs activities) were tested by four-factor ANOVA according to a model (Sudderth et al., 2005). A split-split plot design with CO_2_ and block was used as the main effects, N was used as the subplot effect, and *O. furnacalis* presence or absence was used as the sub-subplot effect (SAS Institute, 2006). The effects of CO_2_ N and block on plant primary metabolism (starch, soluble sugar, and N content and TNC:N ratio) and *O. furnacalis* performance (trypsin, total protease, amylase, lipase, catalase, POD, and SOD activities; and larvae, pupal stage duration, food consumption, MRGR, ECI, ECD, AD metrics) were tested by three-factor ANOVA using a split-plot design with CO_2_ and block as the main effects and N as the subplot effect.

## Results

### C and N Primary Metabolism in Maize

**FIGURE 1:**
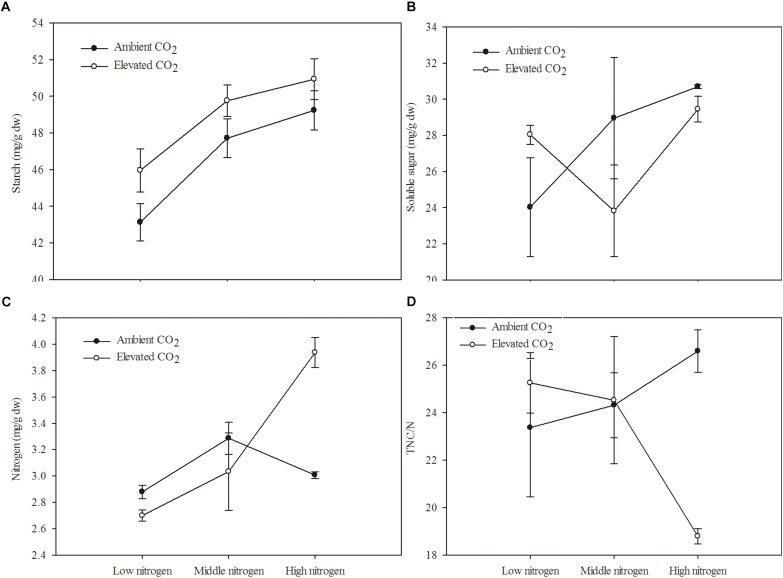
Chemical components of maize grown under different levels of CO_2_ and nitrogen fertilization conditions (mean ± SE, *n* = 3). **(A)** Starch, **(B)** soluble sugar, **(C)** nitrogen (N) and **(D)** total non-structural carbohydrates (TNC)/N.

The C and N primary metabolism of maize was influenced by eCO_2_ and increased N fertilization (Table 2 and Figures 1A–D). Relative to the ambient CO_2_ treatment, the starch content of maize was significantly increased by 4.69% in plants grown under eCO_2_ (Table 2 and Figure 1A). Relative to the low N fertilization treatment, plants grown with middle and high N fertilizations showed a significant increase in the starch content (9.38 and 12.41%, respectively; Table 2 and Figure 1A), and N content (9.38 and 12.42%, respectively; Table 2 and Figure 1C). However, eCO_2_ and increased N fertilization did not influence the soluble sugar content and TNC:N ratio of maize, while the interaction between them was significant on N content and TNC:N ratio (Table 2 and Figure 1B,D).

**Table 2:** Summary of ANOVA results for effects of elevated CO_2_ and increased nitrogen fertilization on chemical components of maize.

Measurement	Treatment	*df*	*F*	*P*
Starch	CO_2_	1	10	0.01
	N	2	23.2	<0.01
	CO_2_^∗^N	2	0.24	0.80
Soluble sugar	CO_2_	1	0.16	0.70
	N	2	1.71	0.24
	CO_2_^∗^N	2	1.8	0.23
Nitrogen	CO_2_	1	3.15	0.11
	N	2	17.83	<0.01
	CO_2_^∗^N	2	16.83	<0.01
TNC/N	CO_2_	1	0.11	0.75
	N	2	1.47	0.29
	CO_2_^∗^N	2	10.45	<0.01

*^∗^TNC, total non-structural carbohydrates; N, low N fertilization level (100 mg N/kg soil mixture), middle N fertilization level (200 mg N/kg soil mixture) and high N fertilization level (300 mg N/kg soil mixture); CO_2_, ambient CO_2_ concentrations (∼380 ppm) and elevated CO_2_ concentrations (∼750 ± 15 ppm); n = 3.*

### *O. furnacalis* Induced Defense-Related Secondary Metabolites in Maize

**FIGURE 2:**
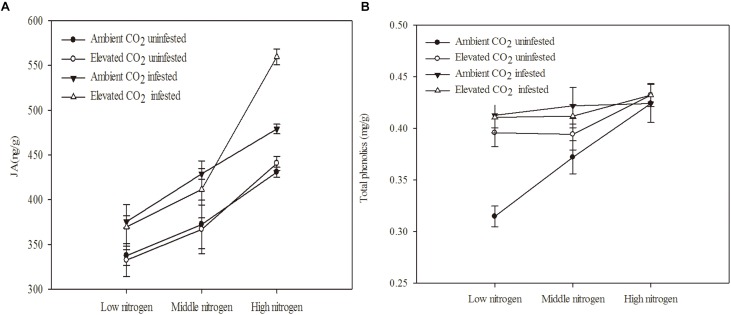
Defensive chemical components of maize grown under different levels of CO_2_ and nitrogen fertilization conditions (mean ± SE, *n* = 3). **(A)** Jasmonic acid (JA) and **(B)** total phenolics.

The chemical defense of maize was significantly influenced by eCO_2_, increased N fertilization and *O. furnacalis* infestation (Table 3 and Figures 2A,B). Relative to ambient CO_2_, the total phenolics content of maize was increased by 5.13% under eCO_2_ (Table 3 and Figure 2B). Relative to low N fertilization, the JA content of maize increased by 11.64 and 34.99% in middle and high N fertilization treatments, while the total phenolics content increased by 11.75% in the high N fertilization (Table 3 and Figure 2A). *O. furnacalis* infestation increased the JA and total phenolics content of maize by 15.06 and 7.69%, respectively, compared with uninfested tissues (Table 3 and Figure 2A,B). Furthermore, the interactions between CO_2_ and infestation and between N and infestation on total phenolics content were significant (Table 3 and Figure 2B).

**Table 3:** Summary of ANOVA results for effects of elevated CO_2_ and increased nitrogen fertilization on chemical defenses of maize.

Measurement	Treatment	*df*	*F*	*P*
JA	CO_2_	1	0.82	0.38
	N	2	48.27	<0.01
	Infestation	1	29.86	<0.01
	CO_2_^∗^N	2	2.94	0.07
	CO_2_^∗^infestation	1	0.86	0.36
	N^∗^infestation	2	1.72	0.20
Total phenolics	CO_2_	1	4.4	0.05
	N	2	9.49	<0.01
	Infestation	1	12.46	<0.01
	CO_2_^∗^N	2	1.66	0.21
	CO_2_^∗^infestation	1	5.23	0.03
	N^∗^infestation	2	3.73	0.04

*^∗^JA, jasmonic acid; N, low N fertilization level (100 mg N/kg soil mixture), middle N fertilization level (200 mg N/kg soil mixture), and high N fertilization level (300 mg N/kg soil mixture); CO_2_, ambient CO_2_ concentrations (∼380 ppm) and elevated CO_2_ concentrations (∼750 ± 15 ppm); n = 3.*

**FIGURE 3:**
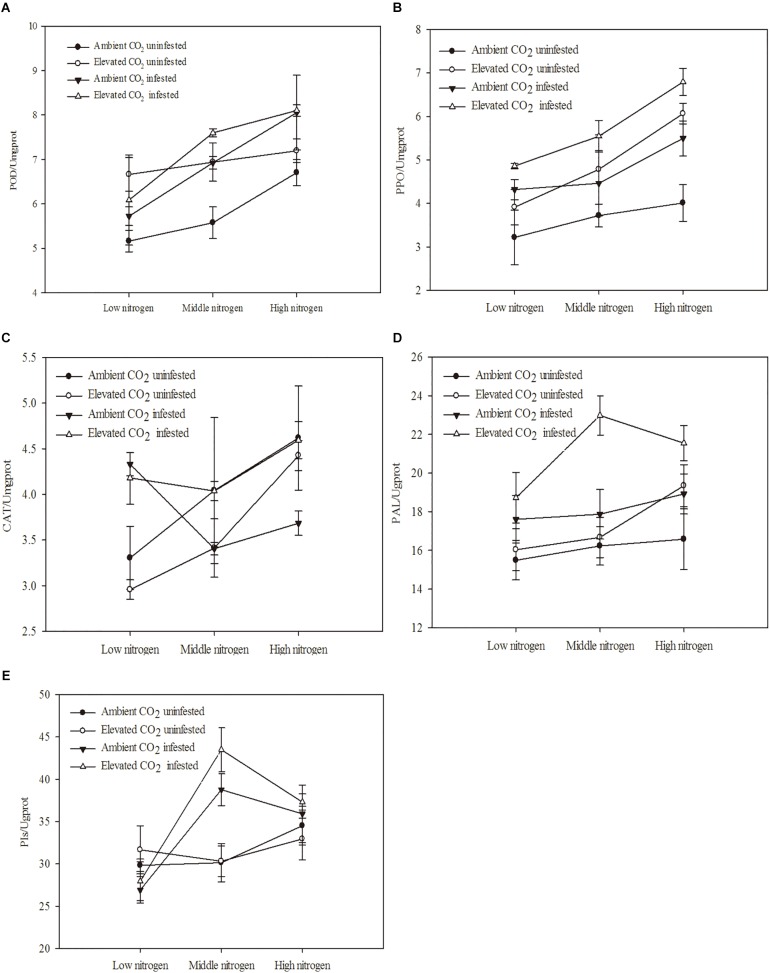
Defensive response of mazie grown under different levels of CO_2_ and nitrogen fertilization conditions (mean ± SE, *n* = 3). **(A)** Peroxidase (POD) activity, **(B)** polyphenol oxidase (PPO) activity, **(C)** catalase (CAT) activity, **(D)** phenylalanine ammonia-lyase (PAL) activity, and **(E)** proteinase inhibitors (PIs).

The defensive enzyme activities of maize were significantly influenced by eCO_2_, increased N fertilization and *O. furnacalis* infestation (Table 4 and Figures 3A–E). Relative to ambient CO_2_, the POD, PPO and PAL activities of the maize were increased by eCO_2_ (Table 4 and Figure 3A,B,D). Relative to low N fertilization, the POD, PAL, CAT, and PIs activities were increased in the middle and high N fertilization treatments, whereas the PPO activities were increased in only the high N fertilization (Table 4 and Figure 3A–E). *O. furnacalis* infestation increased the POD, PPO, PAL, and PIs activities of maize, compared with the uninfested tissues (Table 4 and Figure 3A–C,E). The interactions between CO_2_ and infestation and between N and infestation on CAT activity, and between N and infestation on PIs were significant (Table 4 and Figure 3C,E).

**Table 4:** Summary of ANOVA results for effects of elevated CO_2_ and increased nitrogen fertilization on defensive response of maize.

Measurement	Treatment	*df*	*F*	*P*
POD	CO_2_	1	7.89	0.01
	N	2	13.41	<0.01
	Infestation	1	7.16	0.02
	CO_2_^∗^N	2	0.8	0.46
	CO_2_^∗^infestation	1	2.04	0.17
	N^∗^infestation	2	1.85	0.18
PPO	CO_2_	1	18.53	<0.01
	N	2	11.53	<0.01
	Infestation	1	13.6	<0.01
	CO_2_^∗^N	2	1.38	0.27
	CO_2_^∗^infestation	1	0.32	0.58
	N^∗^infestation	2	0.17	0.84
CAT	CO_2_	1	0.04	0.84
	N	2	5.82	0.03
	Infestation	1	1.92	0.18
	CO_2_^∗^N	2	0.99	0.39
	CO_2_^∗^infestation	1	5.82	0.03
	N^∗^infestation	2	6.54	<0.01
PAL	CO_2_	1	9.48	<0.01
	N	2	3.42	0.05
	Infestation	1	17.83	<0.01
	CO_2_^∗^N	2	0.87	0.43
	CO_2_^∗^infestation	1	1.55	0.22
	N^∗^infestation	2	0.64	0.54
PIs	CO_2_	1	1.03	0.32
	N	2	11.47	<0.01
	Infestation	1	7.82	0.01
	CO_2_^∗^N	2	0.35	0.71
	CO_2_^∗^infestation	1	0.8	0.38
	N^∗^infestation	2	10.84	<0.01

*^∗^PPO, polyphenol oxidase; POD, peroxidase; CAT, catalase; PAL, phenylalanine ammonia-lyase, PIs, proteinase inhibitors; N, low N fertilization level (100 mg N/kg soil mixture), middle N fertilization level (200 mg N/kg soil mixture), and high N fertilization level (300 mg N/kg soil mixture); CO_2_, ambient CO_2_ concentrations (∼380 ppm) and elevated CO_2_ concentrations (∼750 ± 15 ppm); n = 3.*

### The Growth and Development of *O. furnacalis*

**FIGURE 4:**
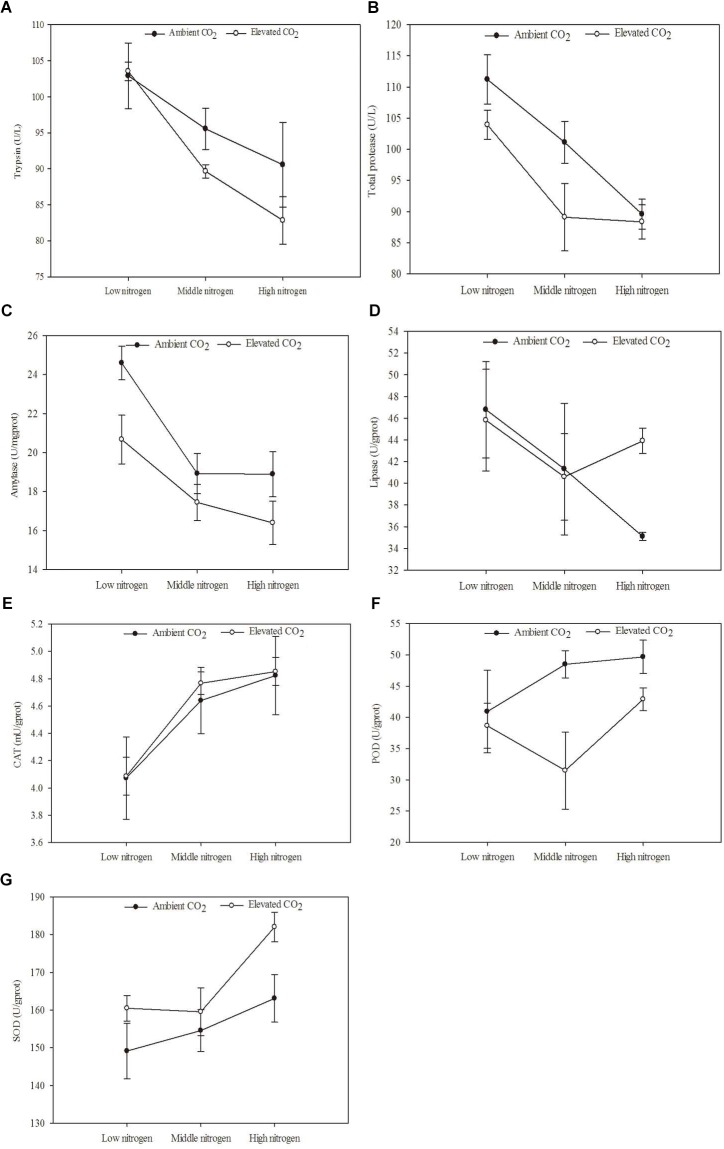
Defense chemical components of *Ostrinia furnacalis* feeding on maize grown under different levels of CO_2_ and nitrogen fertilization conditions (mean ± SE, *n* = 3). **(A)** Trypsin activity, **(B)** total protease activity, **(C)** amylase activity, **(D)** lipase activity, **(E)** catalase (CAT) activity, **(F)** peroxidase (POD) activity, and **(G)** superoxide dismutase (SOD) activity.

The digestive and protective system enzyme activities varied for *O. furnacalis* feeding on maize grown under different levels of CO_2_ and N fertilization (Table 5 and Figures 4A–G). Compared with ambient CO_2_, amylase activity decreased and SOD activity increased for *O. furnacalis* feeding on maize grown under eCO_2_ (Table 5 and Figure 4C,G). Compared with low N fertilization, the trypsin, total protease and amylase activities decreased for *O. furnacalis* feeding on maize grown in the middle and high N fertilization treatments, whereas the CAT and SOD activities increased for *O. furnacalis* feeding on maize grown at high N fertilization and two increasing N fertilization treatments, respectively (Table 5 and Figure 4A–C,E).

**Table 5:** Summary of ANOVA results for effects of elevated CO_2_ and increased nitrogen fertilization on digestive and protective system enzymes of *Ostrinia furnacalis*.

Measurement	Treatment	*df*	*F*	*P*
Trypsin	CO_2_	1	2.78	0.13
	N	2	13.86	<0.01
	CO_2_^∗^N	2	0.95	0.43
Total protease	CO_2_	1	4.09	0.08
	N	2	10.48	<0.01
	CO_2_^∗^N	2	0.85	0.46
Amylase	CO_2_	1	7.55	0.02
	N	2	10.88	<0.01
	CO_2_^∗^N	2	0.55	0.6
Lipase	CO_2_	1	0.67	0.43
	N	2	2.02	0.19
	CO_2_^∗^N	2	1.23	0.34
CAT	CO_2_	1	0.1	0.76
	N	2	7.05	0.02
	CO_2_^∗^N	2	0.04	0.96
POD	CO_2_	1	4.23	0.07
	N	2	1.02	0.40
	CO_2_^∗^N	2	1.07	0.39
SOD	CO_2_	1	6.86	0.03
	N	2	6.19	0.02
	CO_2_^∗^N	2	0.81	0.48

*^∗^CAT, catalase; POD, peroxidase; SOD, superoxide dismutase, N, low N fertilization level (100 mg N/kg soil mixture), middle N fertilization level (200 mg N/kg soil mixture), and high N fertilization level (300 mg N/kg soil mixture); CO_2_, ambient CO_2_ concentrations (∼380 ppm) and elevated CO_2_ concentrations (∼750 ± 15 ppm); n = 3.*

**FIGURE 5:**
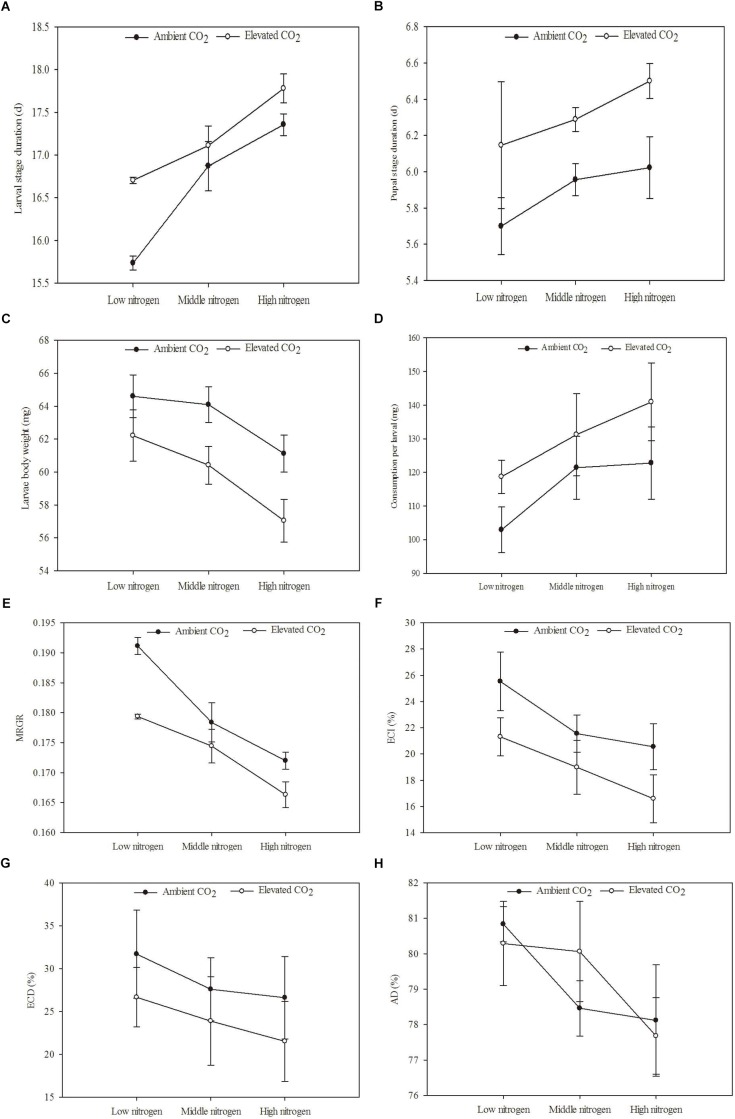
Development and digestibility of *O. furnacalis* feeding on mazie grown under different levels of CO_2_ and nitrogen fertilization conditions (mean ± SE, *n* = 3). **(A)** Larvae stage duration, **(B)** pupal stage duration, **(C)** larvae body mass, **(D)** food consumption, **(E)** mean relative growth rate (MRGR), **(F)** efficiency of conversion of ingested food (ECI), **(G)** efficiency of conversion of digested food (ECD), and **(H)** approximate digestibility (AD).

The development and digestibility of *O. furnacalis* were changed after feeding on maize grown under different levels of CO_2_ and N fertilization (Table 6 and Figures 5A–H). Relative to ambient CO_2_, eCO_2_ extended the duration of the larval and pupal stage by 3.99 and 7.13%, respectively; reduced larval body mass by 5.64%; and decreased MRGR, ECI, and ECD by 4.15, 18.89, and 19.23%, respectively (Table 6 and Figure 5A–C,E,F). Relative to low N fertilization, the middle and high N fertilization treatments extended the larval stage duration by 4.75 and 8.32%, and decreased MRGR by 4.32 and 9.52%, respectively; in the high N fertilization, the larval body mass and ECI were decreased by 7.31 and 26.12%, respectively (Table 6 and Figure 5A,C,E,F).

**Table 6:** Summary of ANOVA results for effects of elevated CO_2_ and increased nitrogen fertilization on development and digestibility of *O. furnacalis*.

Measurement	Treatment	*df*	*F*	*P*
Larvae stage duration	CO_2_	1	15.14	<0.01
	N	2	30.97	<0.01
	CO_2_^∗^N	2	2.43	0.15
Pupal stage duration	CO_2_	1	8.02	0.02
	N	2	1.78	0.23
	CO_2_^∗^N	2	0.09	0.91
Body mass	CO_2_	1	7.44	0.03
	N	2	4.36	0.05
	CO_2_^∗^N	2	0.17	0.85
Food consumption	CO_2_	1	5	0.06
	N	2	3.73	0.07
	CO_2_^∗^N	2	0.14	0.87
MRGR	CO_2_	1	16.21	<0.01
	N	2	27.64	<0.01
	CO_2_^∗^N	2	1.8	0.23
ECI	CO_2_	1	6.89	0.03
	N	2	4.33	0.05
	CO_2_^∗^N	2	0.14	0.87
ECD	CO_2_	1	5.81	0.04
	N	2	2.48	0.15
	CO_2_^∗^N	2	0.05	0.95
AD	CO_2_	1	0.06	0.81
	N	2	3.56	0.08
	CO_2_^∗^N	2	0.74	0.51

*^∗^MRGR, mean relative growth rate; ECI, efficiency of conversion of ingested food; ECD, efficiency of conversion of digested food; AD, approximate digestibility, N, low N fertilization level (100 mg N/kg soil mixture), middle N fertilization level (200 mg N/kg soil mixture), and high N fertilization level (300 mg N/kg soil mixture); CO_2_, ambient CO_2_ concentrations (∼380 ppm) and elevated CO_2_ concentrations (∼750 ± 15 ppm); n = 3.*

## Discussion

Both eCO_2_ and increased N fertilization increased the starch content of the maize in this experiment. A previous study found that the C_4_ photosynthesis in maize was enhanced by increasing N nutrition under eCO_2_ (Cousins and Bloom, 2003), thus the accumulation of photosynthetic products (carbohydrate) might increase in C_4_ plants. The eCO_2_ did not reduce the N content. However, increased N fertilization enhanced the N content in this experiment. Thus, eCO_2_ and increased N fertilization increased C and N primary metabolites, although the combined effects of eCO_2_ and increased N fertilization did not influence the TNC:N ratio. However, our previous results from a different study indicate that eCO_2_ decreases the N content and increases the TNC:N ratio in maize. In this experiment, the altered response of N content to eCO_2_ may be due to the close interaction between CO_2_ and N fertilization (*P* < 0.05, Pal et al., 2005). In other words, increased N fertilization could offset the decreasing N content of maize grown under eCO_2_.

Numerous experiments have shown that defensive metabolisms of plants are influenced by eCO_2_, increased N fertilization, or insect infestation (Lou and Baldwin, 2004; Guo et al., 2012; Zavala et al., 2013; Guo et al., 2018; Lu et al., 2018). However, relatively little is known about their combined effects on the defensive metabolism of plants. In this study, defensive metabolisms of maize were enhanced by eCO_2_, increased N fertilization and insect infestation. We observed that the eCO_2_, increased N fertilization or insect infestation effects were additive, but there were few interactions between factors. The accumulation of JA level and activation of antioxidative enzymes (POD, PPO, and CAT) and PIs play an important role in regulating chewing-induced resistance of plants to insects (Scott et al., 2010; Nahar et al., 2011; Kawazu et al., 2012; Kerchev et al., 2012; Wang and Wu, 2013; War et al., 2015). In this study, both eCO_2_ and increased N fertilization improved defensive enzymes (POD, PPO, and PAL) activities, and increased N fertilization also improved JA level, CAT, and PIs activities. Similarly, a previous study found that the defensive enzyme activities in maize were increased under eCO_2_ or increased N fertilization (Zhang et al., 2007; Zhao et al., 2008). Lou and Baldwin (2004) indicated that JA levels decreased in plants grown with low N fertilization, which was due to the decreased expression levels of JA-related genes in plants grown in low N fertilization. Our observations may be explained by sufficient CO_2_ and N driving the maize metabolites, which enahnced the JA level and defensive enzymes activities at the same time (Nunes-Nesi et al., 2010). Like other chewing insects, *O. furnacalis* infestation can also induce the defensive metabolism of maize (Guo et al., 2018). In the present study, *O. furnacalis* infestation increased JA level, total phenolic content and defensive enzyme (POD, PPO, PAL, and PIs) activities even under eCO_2_ and increased N fertilization conditions, i.e., the maize defensive response still exist under eCO_2_ and increased N fertilization. The activity of PAL, which is a principle enzyme involved in a rate-limiting step in phenolic biosynthesis, could be induced by some enviromental factors (Hartley et al., 2000; Zhao et al., 2009). In this study, PAL activity was enhanced by increased N fertilization and *O. furnacalis* infestation, which subsequently raised the total phenolic content in these treatments. At the same time, there is a positive interaction between *O. furnacalis* infestation and CO_2_ or N fertilization on the total phenolics content in maize plants. We conclude that the defensive metabolism of maize plants grown under elevated CO_2_ and higher N fertilization could be enhanced, especially during *O. furnacalis* infestation.

Plant proteinase inhibitors (PIs) are able to reduce the feeding fitness of chewing insects by suppressing insect gut proteases (Govind et al., 2010). The results in this experiment also indicated that increased N fertilization increased PIs activity in maize, and accordingly, increased N fertilization decreased the activities of *O. furnacalis* digestive enzymes (trypsin, total protease, and amylase). The eCO_2_ also decreased amylase activity, while both eCO_2_ and high N fertilization increased protective enzyme activities. Thus, *O. furnacalis* digestive ability was decreased and the defensive response was enhanced during feeding on maize plants grown under eCO_2_ and increased N fertilization.

Both eCO_2_ and increased N fertilization extended the larvae development time; and decreased the MRGR, body mass and ECI of *O. furnacalis*. These observations could be explained by the results above, due to the fact that TNC:N ratio did not changed, but the defensive metabolism of maize plants was increased by eCO_2_ and increased N fertilization. Thus, the increased defensive metabolism subsequently may increase *O. furnacalis* defensive response, slowed growth and decreased food digestibility and utilization (Xie et al., 2013; Guo et al., 2018; Lu et al., 2018). Henn and Schopf (2001) demonstrated increased consumption and decreased ECI for *Lymantria dispar* fed on trees grown under ambient CO_2_ and low N fertilization. Considering our results, the different effects of eCO_2_ and increased N fertilization on ECI may be due to the N availability and the level of defensive metabolism for host species.

## Conclusion

Our data demonstrate that eCO_2_ and increased N fertilization increased C and N primary metabolites. The combined effects of eCO_2_ and increased N fertilization did not influence the TNC:N ratio, because increased N fertilization could offset the decreasing N content of maize grown under eCO_2_. The resistance-related secondary metabolites (with or without *O. furnacalis* induced) in maize were enhanced by eCO_2_ and increased N fertilization, which increased the *O. furnacalis* defensive response, slowed its growth; and decreased its food digestibility and utilization. Thus, increased N fertilization will increase starch and N accumulation, do not influence the TNC:N ratio. But increased N fertilization promote resistance of maize to *O. furnacalis* feeding, which may decrease the fitness of *O. furnacalis* to its host in future eCO_2_ scenarios.

Furthermore, the metabolic mechanisms of plants and insects may vary with plant and insect species, the insect fitness over multiple generations to host plant and insect enemy behavior may also change under eCO_2_ and increased N fertilization. The optimal N application rates should be employed to improve plant growth and insect population control under eCO_2_. Thus, more research will be needed to elucidate the effects of eCO_2_ and increased N fertilization on interaction of plant-insect, which will help to predict the plant damage in agroecosystem.

## Author Contributions

HuX wrote the manuscript. HaX designed the experiments. HaX and SW performed the experiments. ZW and KH provided the insect, reagents, and materials.

## Conflict of Interest Statement

The authors declare that the research was conducted in the absence of any commercial or financial relationships that could be construed as a potential conflict of interest.
